# Impacts of life-events on sitting, TV viewing and computer use among women from disadvantaged neighbourhoods

**DOI:** 10.1186/s12889-022-14190-w

**Published:** 2022-09-24

**Authors:** Minakshi Nayak, Karen Wills, Megan Teychenne, Verity Cleland

**Affiliations:** 1 Menzies Institute for Medical Research, University of Tasmania, Hobart, TAS Australia; 2 Deakin University, Institute for Physical Activity and Nutrition (IPAN), School of Exercise and Nutrition Sciences, Geelong, Australia

**Keywords:** Sitting, Sedentary behaviour, Women, Low socioeconomic position

## Abstract

**Background:**

Little is known about how life events such as changes in parental or employment status influence sedentary behaviour (SB). Women from disadvantaged neighbourhoods are at particular risk of poor health, therefore, in this population group this study aimed to determine between changes in parental and employment status with sitting, television viewing (TV), and computer time.

**Methods:**

Women (18–45 years) from socioeconomically disadvantaged neighbourhoods self-reported their employment status, number of children, sitting, TV, and computer time [(baseline (*n* = 4349), three (*n* = 1912) and 5 years (*n* = 1560)]. Linear (sitting) and negative binomial (TV and computer time) multilevel models adjusted for confounders were used to estimate the SB association with changes in life events.

**Results:**

Compared to women who never had children during the study period, less sitting and computer time was observed for women when number of children remained unchanged, had their first child or additional child, and fewer children (< 18 years). Less TV was observed for women when number of children remained unchanged.

Compared to women who remained employed full-time during the study period, sitting and computer time decreased among women when they decreased or increased their working hours or when remained employed part-time/not working. TV time increased among women when they decreased their working hours.

**Conclusion:**

Among women, declines in SB were observed amongst those experiencing life events. Interventions to decrease SB may consider targeting women with no children, and future research should further explore how changes in employment type (e.g., non-manual to manual jobs) impact SB.

**Supplementary Information:**

The online version contains supplementary material available at 10.1186/s12889-022-14190-w.

## Background

Sedentary behaviour (SB) is any waking behaviour (e.g., sitting, reclining or lying) characterized by low energy expenditure [≤1.5 metabolic equivalents (MET)] [1]. It is recognised as harmful for health [2], with those of lower socioeconomic position (SEP) at greater risk of poor health and a sedentary lifestyle [3]. Studies have consistently shown women are less active than men and the lower SEP population have a lower level of leisure time physical activity [4–6]. Life events such as the onset of parenthood, joining the workforce or changes in career have shown an impact on health behaviours such as physical activity and diet, particularly in women [7, 8]. Therefore, it is reasonable to expect that sitting, television (TV) viewing, computer use will fluctuate in response to life events, such as having children [9], resulting in deviations from their usual or prior patterns of SB.

Reviews of studies have highlighted the lack of longitudinal studies investigating the impact of life events and SB [10, 11]. One self-reported study using the Australian Longitudinal Study on Women’s Health (ALSWH) data reported that motherhood/having a baby was associated with decreases in sitting time [9], supported by findings from longitudinal studies using device-based measures of sedentary time [12]. The ALSWH also identified that joining the workforce and increases in income were associated with decreases in sitting time while returning to study and job loss were associated with increases in sitting time [9]. The Young Finns study reported that becoming unemployed increases TV viewing among women [13]. Although a systematic review study comparing self-reported and device measured SB highlighted self-report measures underestimate sedentary time compared to device-based measure by 1.74 hours/day [14]. There is only one study examining life-events and SB using device-based measures and showed a decrease in sedentary time over 12 months in women who became mothers for first and second time compared to women without children [12]. Our work among women from socioeconomically disadvantaged neighbourhoods has shown an increase in total sitting and computer time in those who are not working, compared to those who are working full-time, and among those with children compared to those with no children [15, 16]. However, parenthood and employment status exposures in this work were examined at baseline only and did not consider how changes in these factors might relate to changes in SB.

While these findings provide important insights, most focus on a single SB, some group life events together so individual effects cannot be determined, and most only examine change over two-time points [9, 13]. They also give little consideration to the inter-relationship between parenthood and employment status among women, which is strong. For example, motherhood can influence employment status by decreasing working hours or leaving the labour market permanently or temporarily to care for children, subsequently affecting sedentary time [17, 18]. Thus, there is the need to simultaneously consider employment status and parenthood status and changes in these factors when investigating their relationship with SB. This study aimed to examine associations between parental status and employment status with sitting, TV viewing, and computer time among women from disadvantaged neighbourhoods.

## Methods

### Participants

The Resilience for Eating and Activity Despite Inequalities (READI) was a prospective cohort study involving women from socioeconomically disadvantaged neighbourhoods of Victoria, Australia [19]. The main aim of the READI study was to investigate the pathways by which socioeconomic disadvantage influence lifestyle choice associated with obesity risk and to explore mechanisms underlying ‘resilience’ to obesity risk in socioeconomic disadvantaged women and children, details of the READI study were published elsewhere [19, 20]. All Victorian suburbs were classified as urban or rural neighbourhoods and ranked using the Socio-Economic Indexes for Areas (SEIFA) developed by the Australian Bureau of Statistics [21, 22]. Neighbourhoods ranked in the bottom SEIFA third were classified as socioeconomically disadvantaged. Randomly 40 urban and 40 rural neighbourhoods were selected from the bottom SEIFA third for sample collection. Using the Australian Electoral Roll (registration is compulsory for Australian citizens aged ≥18 years), 150 women aged between 18 and 45 years from each of the 80 neighbourhoods were randomly selected and invited by mail to participate. The READI study was initiated in 2007–08, and 4349 women returned complete surveys. The first follow-up data was collected in 2010–11, and responses were received from 1912 participants. The second follow-up was conducted in 2012–13, and surveys were received from 1560 participants.

### Outcomes

#### Sitting time

The International Physical Activity Questionnaire-long version (IPAQ-L) was used to measure total sitting time on weekdays and weekends in the past week [e.g., “During the last 7 days, how much time did you usually spend sitting (at work, at home or leisure such as TV viewing) on a weekday day?”] [23]. Participants self-reported the usual time they spent sitting each day during week and on weekends in the past week. Total hours spent sitting per week were calculated by multiplying daily sitting on weekdays by five and on weekends by two. These values were summed then divided by 60 to estimate the total minutes sitting per week. IPAQ-L for sitting time has demonstrated good test-retest reliability (Spearman rho values above 0.70) and acceptable validity (correlation coefficient (ICC) of 0.30 with accelerometer) [23, 24]. As per the IPAQ protocol [25], assuming 8 hrs sleep in 24 hours [26], values greater than 16 hours/day for sitting time were considered implausible and treated as missing.

#### TV viewing and computer time

Participants reported the usual daily TV viewing and computer time on both weekdays and weekends in the past week (e.g., “Of your total sitting time, during the last 7days, how much time did you usually spend sitting watching TV on a weekday?”) and computer time (e.g., “Of your total sitting time, during last 7 days, how much time did you usually spend sitting at the computer on a weekday?”) [27]. Weekly TV viewing and computer time (hrs/week) were estimated by multiplying daily weekday duration by five and weekend duration by two, then summing these values (separately for each behaviour) and dividing by 60. Both TV viewing [ICC = 0.82 (95% CI; 0.75, 0.87)] and computer time [ICC = 0.62 (95% CI; 0.48, 0.73)] have acceptable test-retest reliability in Australian adults and have validity with a 3-day SB log (TV viewing: Spearman rank-order correlation = 0.30, *p* < 0.01; computer use: Spearman rank-order correlation = 0.60, p < 0.01) [28]. Observations of > 16 hours/day for either TV viewing or computer time were considered implausible and treated as missing based on the assumption described above. In this study, TV and computer time were investigated separately as distinct SB. TV viewing is a leisure time SB (discretionary) and hence more amenable to interventions whereas computer could be influenced by occupation (non-discretionary) [29].

### Exposures

#### Life events

Employment status and number of children (aged < 18 years) living at home were modelled as time-varying predictors [30]. At each time point, participants reported employment status (full-time, part-time, not working) and number of children (aged < 18 years) living at home (none, one, two, three or more). To allow estimation of between-person and within-person associations, we computed two components for the life-events variables. Parental status was coded as a binary variable indicating whether a participant had children < 18 years living with them at any time during the study period (between-person effect): 0 = No, 1 = Yes; and a categorical variable to capture changes in parental status at each time point (within-person effect): 0 = never had children, 1 = number of children remained unchanged during the study period, 2 = first or additional children, 3 = fewer children (aged < 18 years) living at home. Employment status was coded as a binary variable indicating whether a participant was employed full-time at any time during the study period (between-person effect): 0 = No, 1 = Yes: and a categorical variable to capture changes in employment status at each time point (within-person effect): 0 = Remained full-time during the study period, 1 = remained part-time or not working, 2 = decreased working hours, 3 = increased working hours.

#### Confounders

Baseline sociodemographic confounders included self-reported age (years), body mass index (BMI) (kg/m^2^, derived from weight (kg) divided by height (m^2^)), area of residence (rural, urban), health status (excellent, very good, good, fair to poor), smoking status (never smoked, used to smoke, smoke occasionally, smoke regularly) and level of education (low = no formal qualification/completed year 10 or equivalent, medium = completed year 12/apprenticeship/diploma, high = completed tertiary education). From previous descriptive studies none of these confounders varied sufficiently over time to warrant inclusion as time varying confounders [15, 16]. Besides, these confounders were identified using a 10% change in estimation criteria when adjusted in models [31].

### Statistical analysis

Data analysis was completed using STATA version 16 [32]. Participant baseline characteristics were summarized by frequency (N) and percentage (%) for categorical data and by the mean (standard deviation) for continuous data.

Sitting time was normally distributed, so linear mixed models were used to estimate within-person and between-person changes in sitting time associated with parental status and employment status changes. Models were estimated using maximum likelihood estimation, and effect estimates are presented as beta-coefficients. TV viewing and computer time were count response variables (positive integer values greater than or equal to zero), and the distributions of both outcomes were over-dispersed (variance greater than the mean). Therefore, mixed-effects negative-binomial regression was used to estimate within-person and between-person association between parental status and employment status and changes in TV viewing and computer time. Effect estimates are presented as exponentiated coefficients, ratios of mean TV viewing and computer time, indicating the magnitude of change for one-unit increase in exposure. Estimates for categorical exposures indicate the magnitude of change for each level of exposure relative to the reference category. For example, an effect estimates of 0.90 for a certain level of exposure would indicate a 10% reduction in outcome relative to the reference level, whilst an estimate of 1.10 would indicate a 10% increase in outcome relative to the reference level.

Separate models were fitted for both life event exposures and each outcome variable. Three models for each exposure (parental change and employment change) and outcome were assessed; the first model was adjusted for time, the second model was adjusted for time, and baseline confounders and the third model were adjusted for time, time-varying covariate, and baseline confounders. All models included a random intercept for each participant to account for individual differences in outcome means. The correlation between the repeated measurements over time was modelled using an auto-regressive residual variance-covariance structure. When this model would not converge, an independent variance-covariance structure with cluster-robust standard errors that allow for correlation among the repeated observations for an individual was specified.

Multiple imputations using the method of chained equation (assuming data were missing at random [33]) were used to address the missing data (incomplete observation and attrition). The imputed model included baseline variables for age, BMI, area of residence, health status, smoking status, education, marital status and variables from three times points for the number of children, employment status and outcomes (sitting, TV viewing and computer time). Exposure variables (change in parental status and change in employment status) were imputed as passive variables. For each outcome, a separate imputation model was run using baseline variables (e.g., age, BMI), variables from three-time points, exposure variables and the outcome; 50 data set were created for every outcome variable [34].

### Sensitivity Analysis

A sensitivity analysis was conducted to compare the results of analysis using multiple imputations with results of analysis using a complete case approach.

## Results

Baseline characteristics of the sample are reported in Table 1. Women who participated at T1 only or T1 and T2 compared to those who were not lost to follow-up were older, non-smokers, had better health, higher education, higher incomes, living in rural areas, working part-time, married, had children, reported sitting less and watched less TV (Supplementary Table 1). There are more women reporting the birth of a first or additional child between T1 and T2 than T2 and T3 and numerous participants reported changing working hours between all time points (Table 2).

**Table 1:** Baseline (2007–08) sociodemographic and health characteristics of participants in the READI study

Variables	***N*** = 4349
Age (years) (Mean/SD)	34.4 (8.1)
BMI (kg/m^2^) (Mean/SD)	26.05 (6.0)
General Health %(N)	
Excellent	9.1 (392)
Very good	34.8 (1508)
Good	41.5 (1799)
Poor or fair	14.6 (631)
Smoking %(N)	
Never smoked	50.2 (2183)
Used to smoke	24.5 (1066)
Smoke occasionally	9.5 (411)
Current smoker	15.8 (684)
Area of residence %(N)	
Urban	46.4 (2016)
Rural	53.6 (2331)
Marital Status %(N)	
Married	65.5 (2829)
Widowed/separated/divorced	8.5 (370)
Never married	26.0 (1122)
Education Level %(N)	
Low	22.1 (946)
Medium	51.7 (2216)
High	26.2 (1120)
Employment status %(N)	
Working full-time	38.1 (1613)
Working part-time	29.4 (1245)
Not working	32.4 (1372)
Number of children %(N)	
None	39.4 (1678)
One	18.5 (787)
Two	25.5 (1086)
Three or more	16.7 (713)
Sitting time (hours/week) (Mean/SD)	40.9 (21.5)
Television (hour/week) (Median/IQR)	16.5 (11–27)
Computer (hour/week) (Median/IQR)	9.5 (2.5–27.5)

Abbreviation: *BMI-* Body mass index, *SD-* Standard deviation, *N (%)-* Number (percentage), IQR- interquartile range

**Table 2:** Changes in parental status and employment status among participants of READI study between baseline and first follow-up (T1 to T2) and first follow-up to second follow-up (T2 to T3)

Exposure variable	From T1 to T2	From T2 to T3
**Change in parental status, %(N)**		
No children	6.1 (81)	5.7 (66)
Number of children remained unchanged	59.2 (781)	70.6 (709)
First child/Additional child/ren	21.9 (290)	8.4 (85)
Few children (aged < 18 years) living at home	12.7 (168)	15.3 (154)
**Change in employment status, %(N)**		
Remained working as full-time	28.2 (511)	31.9 (473)
Remained working as part-time/ not working	38.0 (689)	40.7 (606)
Increased their working hours	19.8 (359)	15.6 (231)
Reduced their working hours	13.9 (252)	11.7 (173)

Abbreviation:*%(N)-* percentage (number)

### Change in parental status and employment status and sitting time

For changes in parental status (full adjusted model), women who had fewer children living at home (aged < 18 years) during the study sat on average 4.4 hours/week less (95% CI: − 6.7, − 2.1) per week than women who did not have children (between-person effect). Within-person changes in parental status were consistently associated with reductions in sitting time; compared to women who did not have children, sitting time was reduced by 4.0 hours/week (95% CI: − 6.5, − 1.4) when the number of children remained unchanged and by 4.2 hours/week (95% CI: − 6.9, − 1.6) when women who had their first child or additional children (Table 3).

**Table 3:** Linear mixed model estimates of between-person and within-person changes in sitting behaviour associated with changes in parental status and employment status over 5 years (2007/08–2011/13)

Sitting time (hours/week)					
	**Model 1**	**Model 2**	**% of Change**	**Model 3**	**% of Change**
	**β (95% CI)**	**β (95% CI)**		**β (95% CI)**	
**Change in parental status**					
Between-person effects					
Never had children	Ref				
Living with children < 18 during study	−6.6 (− 8.9, − 4.4)***	−6.1 (− 8.3, − 3.8)***	−7.6%	− 4.4 (− 6.7, − 2.1)***	−27.9%
Within-person effects					
Never had children	Ref				
Number of children remained unchanged	−4.6 (−6.9, − 2.3)***	−4.1 (− 6.4, − 1.7)**	−10.9%	−4.0 (− 6.5, − 1.4)**	− 2.4%
First child/ additional children	−4.1 (− 6.7, − 1.6)**	−4.2 (− 6.7, − 1.6)**	2.4%	−4.2 (− 6.9, − 1.6)**	0.0%
Fewer children < 18 yrs. living at home	−3.6 (− 6.8, −0.2)*	− 2.7 (− 6.1, 0.7)	−25.0%	− 2.8 (− 6.4, 0.8)	3.7%
**Change in employment status**					
Between-person effects					
Employed full-time during the study period	Ref				
Not employed full-time during study period	−5.6 (− 7.1, −4.2)***	−5.1 (− 5.8, −2.3)***	−8.9%	−3.8 (− 5.7, − 2.1)***	− 25.5%
Within-person effects					
Remained full-time during the study period	Ref				
Remained part-time/notworking	−0.3 (−2.1, 1.4)	− 0.2 (− 2.1, 1.5)	−33.3%	−1.0 (− 2.9, 0.9)	400.0%
Increased working hours	−2.5 (−4.5, − 0.5)*	−2.4 (− 4.4, − 0.4)*	−4.0%	−2.4 (− 4.5, − 0.3)*	0.0%
Reduced working hours	− 2.6 (− 4.8, − 0.3)*	−2.5 (− 4.7, − 0.2)*	− 3.8%	−2.2 (− 4.5, − 0.1)*	− 12.0%

Abbreviation: *β-* Beta coefficient, *CI-* Confidence interval, ***p-value = < 0.001, **p-value = < 0.01, *p-value = < 0.05, % of change = difference between the models reported in percentage

Model 1: Change in parental status and sitting; adjusted for time

Model 2: Change in parental status and sitting; adjusted for time, baseline age, education, health status and area of residence

Model 3: Change in parental status and sitting; adjusted for time, change in employment (Between Person/Within Person), and baseline age, education, health status and area of residence

Model 1: Change in employment status and sitting; adjusted for time

Model 2: Change in employment status and sitting; adjusted for time, baseline age, education and area of residence

Model 3: Change in employment status and sitting; adjusted for time, change in the number of children (Between Person/Within Person), baseline age, education, health status and area of residence

For employment status (full adjusted model), women who were not employed full-time during the study period sat an average 3.8 hours/week less (95% CI: − 5.7, − 2.1) compared to women who were employed full-time during the study (between-person effect). Within-person changes in employment status were associated with reductions in sitting time; compared to women who were employed full-time, sitting time was reduced by 2.4 hours/week (95% CI: − 4.5, − 0.3) when women increased their working hours and by 2.2 hours/week (95% CI: − 4.5, − 0.1) when women decreased their working hours (Table 3).

### Change in parental and employment status and TV viewing

For changes in parental status (fully adjusted model), women living with children < 18 years at any time during the study watched 16% (95% CI: 9, 22%) less TV per week on average, than women who did not have children (between-person effect). Within-person changes in parental status were also associated with a reduction in TV viewing; compared to women who did not have children, TV viewing was reduced by 10% per week (95% CI: 1, 18%) when the number of children remained unchanged (Table 4).

**Table 4:** Mixed-effects negative binomial regression estimates of between-person and within-person changes in TV viewing associated with changes in parental status and employment status over 5 years (2007/08–2011/13)

TV viewing (hours/week)					
	Model 1	Model 2	% of Change	Model 3	% of Change
	**IRR (95% CI)**	**IRR (95% CI)**		**IRR (95% CI)**	
**Change in parental status**					
Between Person effects					
Never had children	Ref				
Living with children < 18 during study	0.87 (0.80, 0.94)***	0.86 (0.78, 0.93)***	−1.1%	0.84 (0.78, 0.91)***	−2.3%
Within Person effects					
Never had children	Ref				
Number of children remained unchanged	0.91 (0.84, 0.99)*	0.89 (0.82, 0.98)*	−2.2%	0.90 (0.82, 0.99)*	1.1%
First child/ Additional children	0.90 (0.81, 1.01)	0.92 (0.83, 1.01)	2.2%	0.93 (0.83, 1.03)	1.1%
Fewer children (aged < 18 yrs) living at home	0.99 (0.87, 1.11)	0.94 (0.83, 1.07)	−5.0%	0.96 (0.84, 1.10)	2.1%
**Change in employment status**					
Between Person effects					
Employed full-time during the study period	Ref				
Not employed full-time during study period	1.00 (0.96, 1.05)	0.99 (93, 1.03)	−1%	1.00 (0.95, 1.05)	1%
Within Person effects					
Remain full-time during the study period	Ref				
Remain part-time/not-working	1.07 (0.99, 1.14)	1.05 (0.98, 1.12)	−1.8%	1.07 (0.99, 1.15)	1.9%
Increased working hours	0.99 (0.92, 1.07)	0.97 (0.90, 1.05)	−2.0%	0.99 (0.92, 1.08)	2.1%
Reduced working hours	1.11 (1.01, 1.21)*	1.09 (1.01, 1.19)*	−1.8%	1.11 (1.03, 1.22)*	1.8%

Abbreviation: *IRR-* ratio of mean, *CI-* Confidence interval, ***p-value = < 0.001, **p-value = < 0.01, *p-value = < 0.05% of change = difference between the models reported in percentage

Model 1: Change in parental status and TV time; adjusted for time

Model 2: Change in parental status and TV time; adjusted for time, baseline age, BMI, education status and smoking status

Model 3: Change in parental status and TV time; adjusted for time, change in employment (Between Person/Within Person), and baseline age, BMI, education status and smoking status

Model 1: Change in employment status and TV time; adjusted for time

Model 2: Change in employment status and TV time; adjusted for time, baseline age, BMI, education status and smoking status

Model 3: Change in employment status and TV time; adjusted for time, change in parental status (Between Person/Within Person) and baseline age, BMI, education status and smoking status

For changes in employment status (fully adjusted model), there was no significant difference in average TV viewing found between women who were not employed full-time, and women employed full-time during the study (between-person effect). However, within-person changes in employment status were associated with differences in TV viewing; compared to women remained full-time employed during the study, TV viewing increased by 11% per week (95% CI: 3, 22%) when women decreased their working hours (Table 4).

### Change in parental status and employment status and computer time

For changes in parental status (fully adjusted model), there was no significant difference in average computer time found between women who were living with children aged < 18 years at any time during the study and women who did not have children (between-person effect). However, within-person changes in parental status were associated with changes in computer time; compared to women who did not have children; computer time was reduced by 22% per week (95% CI: 10, 33%) for women whose number of children remained unchanged, by 25% per week (95% CI: 12, 36%) when women had their first child or additional children and by 20% per week (CI 95% CI: 3, 34%) when living with fewer children (aged < 18 years) (Table 5).

**Table 5:** Mixed-effects negative binomial regression estimates of between-person and within-person changes in computer time associated with changes in parental status and employment status over 5 years (2007/08–2011/13)

Computer time (hours/week)					
	**Model 1**	**Model 2**	**% of**	**Model 3**	**% of chaChange**
	**IRR (95% CI)**	**IRR (95% CI)**	**change**	**IRR (95% CI)**	**change**
**Change in parental status**					
Between Person effects					
Never had children	Ref				
Living with children under 18 during study	0.75 (0.67, 0.85)***	0.84 (0.75, 0.96)*	12%	0.91 (0.80, 1.03)	8.3%
Within Person effects					
Remain with no children	Ref				
Number of children remain unchanged	0.74 (0.64, 0.84)***	0.78 (0.69, 0.90)**	5.4%	0.78 (0.67, 0.90)**	0%
First child/ Additional children	0.77 (0.66, 0.90)**	0.77 (0.66, 0.90)**	0%	0.75 (0.64 0.88)**	−2.6%
Fewer children < 18 yrs. living at home	0.76 (0.64, 0.91)**	0.83 (0.69, 1.01)	9.2%	0.80 (0.66, 0.97)*	−3.6%
**Change in employment status**					
Between Person effects					
Employed full-time during the study period	Ref				
Not employed full-time during the study period	0.70 (0.66, 0.75)***	0.73 (0.68, 0.78)***	4.3%	0.79 (0.73, 0.85)***	8.2%
Within Person effects					
Remain full-time	Ref				
Remain part-time/not working	0.81 (0.74, 0.88)***	0.82 (0.75, 0.90)***	1.2%	0.76 (0.68, 0.84)***	−7.3%
Increased working hours	0.87 (0.80, 0.95)**	0.89 (0.81, 0.98)**	2.3%	0.88 (0.80, 0.97)*	−1.2%
Reduced working hours	0.66 (0.59, 0.75)***	0.67 (0.60, 0.75)***	1.5%	0.67 (0.59, 0.75)***	0%

Abbreviation: *IRR-* ratio of mean, *CI-* Confidence interval, ***p-value = < 0.001, **p-value = < 0.01, *p-value = < 0.05, % of change = difference between the models reported in percentage

Model 1: Change in parental status and computer time; adjusted for time

Model 2: Change in parental status and computer time; adjusted for time, baseline age, education status and area of residence

Model 3: Change in parental status and computer time; adjusted for time, change in employment (Between Person/Within Person), and baseline age, education status and area of residence

Model 1: Change in employment status and computer time; adjusted for time

Model 2: Change in employment status and computer time; adjusted for time, baseline age, education status and area of residence

Model 3: Change in employment status and computer time; adjusted for time, change in parental status (Between Person/Within Person), baseline age, education status and area of residence

For employment status (full adjusted model) women who were not employed full-time were estimated to spend 21% (95% CI: 16, 27%) less time on computer per week compared to women employed full-time during the study (between-person effect). Within-person changes in employment status were associated with reductions in computer time; compared to women who were employed full-time during the study, computer time was reduced by 24% per week (95% CI: 16, 32%) when remained part-time or not working, by 12% per week (95% CI: 3, 20%) when working hours increased, and by 33% per week (95% CI: 25, 41%) when working hours decreased (Table 5).

### Sensitivity analysis

Sensitivity analyses (Supplementary Tables 2, 3 and 4) using complete case data were comparable to the results using multiple imputations for sitting, TV viewing and computer time. There were small to moderate differences in effect size (with results from the imputed dataset being marginally attenuated), but the patterns were same. Changes in inference were noticed for the between-person effect on sitting and computer time for a change in parental status; within-person effect on TV viewing for the number of children remained unchanged (Model 1).

## Discussion

This study aimed to estimate associations between change in parental and employment status with sitting, TV viewing, and computer time among women living in disadvantaged neighbourhoods. Our study demonstrates that between-person and within-person changes in parental status and changes in employment status are associated with changes in sitting, TV and computer time. Compared to women with no children, those who experienced a change in their parental status (given birth to their first child or additional child/ren, having fewer children < 18 years living at home with them) or who had the same number of children showed a significant decrease in their sitting and computer time. Also, compared to women with no children, those who had the same number of children watched less TV. Compared with those remaining in full-time work, lower sitting was observed in women who increased or decreased working hours. Likewise, compared with those remaining in full-time work, lower computer time was observed in women who remained working part-time/not working or increased or decreased working hours. However, compared to women who remained in full-time work, higher TV viewing was observed in those who decreased their work hours.

### Change in parental status and SB

Both between-person and within-person differences in parental status were associated with decreased sitting, TV viewing and computer time. A further reduction for between-person differences in parental status and sitting (− 27%) and TV viewing (− 2.3%) was seen after adjustment, indicating an independent effect. This is consistent with previous prospective studies that reported the birth of a child in young women was associated with decreased sedentary [9, 12] and TV time [13], but this is the first study to examine associations with computer time. This within-person reduction in SB among first time or subsequent child mothers might be due to the care needed for young infants, which likely involves regular light-intensity activities that displace sedentary time. Compared to women who had no children during the study, sitting time was less for women when the number of children remained unchanged and when living with fewer children (aged < 18 years), suggesting that the care and support of children per se, not just young children require more light intensity activities. In previous literature, light intensity activity (< 3 MET) which include casual walking, household chores such as cooking, laundry [35] was positively and sedentary time was negatively associated with parenthood in cross-sectional studies using device-based measures [36, 37]. Our longitudinal findings could suggest parenthood in women may not just decrease their sedentary time but also increases light intensity activities which has numerous health benefits such as reduced metabolic risk [38] and increased physical health and well-being [39]. Further, a mother’s involvement in various recreational activities with young children can be physically demanding (e.g., taking children to parks or activity centres, playing with children at home), which may decrease sitting time. Reductions in computer time among women whose number of children remained unchanged and when they had a first child or additional children during study may indicate that to fulfil motherhood duties, fewer hours are spent in work and leisure activities that require computer use. These results suggest that giving birth to a child or having young children at home may protect against sedentary time. Therefore, intervention studies targeting reductions in SB may warrant a focus on women without children.

### Change in employment status and SB

When the time-varying covariate and confounders were adjusted, there was a further reduction in between-person differences found for employment status and sitting (− 25.5%). In contrast, the between-person effect for employment status and computer time increased (8.2%) after adjustment (but between person effect show no change in direction of association). These results indicate associations between change in employment status and sitting and computer time are independent of change in parental status. Compared to their full-time employed counterparts, women who decreased their work hours sat less and women who remained in part-time work, not working or decreased work hours reported less computer time. These findings are not surprising since previous research shows the highest levels of sitting time [10, 16] and computer use [15] amongst full-time workers. That women who increased working hours reported less sitting and computer time than women who remained employed full-time could be due to employment in less sedentary jobs. Women in this study were predominantly with low to medium levels of education (74%), so they may be more likely to engage in manual or physically demanding jobs (e.g., hairdresser, cleaner) than women with higher levels of education [40]. Given participant occupation type (e.g., manual or non-manual/desk job) data was not available, we are unable to confirm this hypothesis. Furthermore, the measures of computer time were not context or domain-specific (e.g., leisure time or occupational computer time), which could explain this unexpected result. When women decreased their working hours, TV viewing increased, consistent with a previous finding where becoming unemployed increased TV viewing [13]. Intervention studies to support women from disadvantaged neighbourhoods who decrease their working hours may be warranted to prevent increases in TV viewing.

When designing interventions for specific population groups, it is important to consider the factors related to that particular group and how these might impact SB. For example, technology-based interventions to reduce SB have shown be efficacious in the general population [41–43] however, little research has investigated the effectiveness of these interventions specifically in socioeconomically disadvantaged populations. A review on activity permissive workstations (e.g., sit-stand desk) has shown this strategy results in reduced occupational sedentary time [44], yet for those in blue (e.g., factory workers) or pink-collar jobs (e.g., caregivers, hairdressers), such intervention strategies may be inappropriate. Thus, recommending one-size-fits-all interventions for women may be ineffective for women living in disadvantaged neighbourhoods. For example, women with no children, who are not working or who decrease work hours over time may be spending more time on sedentary leisure pursuits (e.g., using social media) rather than sitting at a desk using the computer for work purposes. Therefore, designing interventions to reduce sedentary time among this group of disadvantaged women may need to be targeted and tailored differently to those who are working more and those with children. This might include strategies targeting reductions in social media use specifically for women not working/at home or those with no children, while strategies for those employed/working might target reductions in workplace sitting.

### Strength and limitations

A key strength of this study was its prospective design, which allowed investigation of and temporal insights into relationships between two major life events and SB over 5 years. Secondly, the sample focused on women from socioeconomically disadvantaged neighbourhoods, a high-risk group for SB and poor health. Another strength was mixed modelling, which included time-varying covariates to adequately capture SB changes over time [45]. Finally, to our knowledge, this was the first study to assess the impact of life events on computer time, an increasingly prominent SB.

Limitations of this study include the low baseline response rate and high attrition, which may affect generalizability of finding. However, the initial response rate of 45% could be considered acceptable amongst this hard to reach population group [46]. Participants remaining in the study were overrepresented by older women (age 36-45 years), yet there was considerable heterogeneity in the baseline characteristics (e.g., 32% not working, 52% with medium and 22% with low education, and 22% obese). Multiple imputations were used to handle missing data, potentially reducing the dropout biasness, and findings using imputed data were comparable to those from complete case analysis. Secondly, this study lacked data on contemporary screen-based behaviour (e.g., smartphone/tablet use) [47]. Therefore, our results may not represent all SB. The self-report nature of surveys increases the chance of bias and recall difficulties, but measures with excellent reliability and acceptable validity were used [23, 28, 48]. However, to avoid over-or under-reporting more device-based measurement on SB are recommended for future studies on life-events. Measures lacked context- and domain-specific information about SB (e.g., occupational versus leisure). There was also a lack of information on employment type (e.g., office workers or manual labour) or working hours, which may have provided further insights into how changing employment status within various professions affect SB.

## Conclusion

This study provides novel insights into the changing patterns of SB in women from socioeconomically disadvantaged neighbourhoods and the life events associated with these changes. The findings suggest that motherhood may be a protective factor against SB; therefore, women without children may be a worthy target group for interventions to reduce sedentary time. Future studies need to consider changes in occupation type (e.g., manual or non-manual) and working hours in relation to SB.

## Supplementary Information

**Additional file 1.**

## Data Availability

The dataset used/or and analysed during the current study are not publicly available (intellectual property issues) but are available from the corresponding author on reasonable request.

## Abbreviations

SB: Sedentary behaviour
MET: Metabolic equivalent
SEP: Socioeconomic Position
TV: Television
ALSWH: Australian Longitudinal Study on Women’s Health
READI: Resilience for Eating and Activity Despite Inequalities
SEIFA: Socio-Economic Indexes for Areas
IPAQ-L: International Physical Activity Questionnaire-long version
BMI: Body mass index
ICC: Intra-correlation coefficient
