# Supplementary material for: Impacts of life-events on sitting, TV viewing and computer use among women from disadvantaged neighbourhoods
Source: BMC Public Health. 2022 Sep 24;22:1816. doi: 10.1186/s12889-022-14190-w (PMC9508715; doi:10.1186/s12889-022-14190-w)
Supplement: Supplementary file 1 — Additional file 1. [file 12889_2022_14190_MOESM1_ESM.docx]

**Supplementary Table 1**.**Comparison of baseline characteristics between participants who completed surveys at three-time points (T1, T2 and T3), participants who completed a survey only at T1, and participants who completed surveys at both T1 and T2**

| **Variables** | **Completed surveys at T1, T2, T3 (n=1560)** | **Completed surveys at T1 and T2 only (n=353)** | **Completed survey at T1 only (n=2347)** |
| --- | --- | --- | --- |
| Age in years, (mean, SD) (range) | 36.2 (7.6) | 33.7 (8.2) (23.5-54.4) | 33.1 (8.3) (21.3- 52.5) |
| BMI kg/m^2^ (mean, SD) | 26.1 (5.8) | 26.1 (6.2) | 26.0 (6.1) |
| Area of residence (N, %) |  |  |  |
| Urban | 623 (40.2) | 142 (40.3) | 1251 (51.7) |
| Rural | 937 (59.8) | 211 (59.6) | 1155 (48.3) |
| General Health (N, %) |  |  |  |
| Excellent | 180 (12.8) | 27 (7.9) | 173 (7.5) |
| Very good | 554 (37.9) | 99 (29.3) | 803 (34.7) |
| Good | 599 (36.7) | 155 (45.7) | 968 (41.9) |
| Poor/Fair | 177 (12.6) | 58 (17.1) | 369 (15.9) |
| Smoking status (N, %) |  |  |  |
| Never smoked | 817 (50.4) | 155 (49.0) | 1212 (49.8) |
| Used to smoke | 414 (23.4) | 94 (23.4) | 558 (22.9) |
| Smoke occasionally | 122 (10.1) | 47 (10.3) | 242 (9.9) |
| Smoke regularly | 207 (17.1) | 57 (17.3) | 421 (17.4) |
| Level of Education (N, %) |  |  |  |
| Low | 331 (21.3) | 81 (21.8) | 534 (21.7) |
| Medium | 755 (48.6) | 191 (53.8) | 1270 (53.8) |
| High | 467 (30.1) | 77 (24.3) | 576 (24.5) |
| Employment status (N, %) |  |  |  |
| Working full-time | 578(37.7) | 116 (33.4) | 919 (39.8) |
| Working part-time | 501 (32.7) | 116 (33.4) | 628 (26.5) |
| Not working | 455 (29.6) | 115 (33.2) | 802 (33.7) |
| Average gross income (N, %) |  |  |  |
| No income | 112 (7.5) | 31 (8.8) | 164 (0.6) |
| $1-699 /week | 589 (39.0) | 193 (52.7) | 1293 (56.8) |
| $700-1499/week | 616 (41.5) | 69 (19.5) | 454 (19.8) |
| $1500 or more/week | 46 (3.1) | 9 (2.0) | 46 (2.0) |
| Don’t know/want to answer | 133 (8.9) | 32 (9.0) | 323 (18.2) |
| Marital Status (N, %) |  |  |  |
| Married | 1128 (72.3) | 241 (68.5) | 1460 (60.4) |
| Separated | 117 (7.8) | 33 (9.4) | 216 (8.8) |
| Never Married | 296 (19.8) | 78 (22.2) | 735 (30.7) |
| Number of children (N, %) |  |  |  |
| None | 540 (36.5) | 110 (31.5) | 1006 (42.5) |
| One | 264 (17.8) | 78 (22.4) | 431 (18.1) |
| Two | 394 (26.6) | 98 (28.1) | 572 (24.2) |
| Three or more | 281 (19.0) | 63 (18.0) | 364 (15.2) |
| Total sitting hrs/week | 40.0 (21.00) | 40.3 (22.2) | 41.6 (21.9) |
| Total TV time hrs/week | 16 (10.5-26) | 17 (11.29) | 17 (11-28) |
| Total Computer time hrs/week | 10 (3-29) | 11 (2.7-30) | 11 (2.8-30) |

Abbreviations: BMI- body mass index, SD- standard deviation, N (%)- number(percentage), T1=2007-08, T2=2010-11, T3=2012-13

**Supplementary Table 2. Linear mixed model estimates of between-person and within-person changes in sitting behaviour associated with changes in parental status and employment status over five years (2007/08-2011/13)**

|  | **Sitting Time (hours/week)** | | |
| --- | --- | --- | --- |
|  | **Model 1** | **Model 2** | **Model 3** |
|  | **β -coefficient (CI)** | **β -coefficient (CI)** | **β -coefficient (CI)** |
| **Change in parental status** |  |  |  |
| Between-person effects |  |  |  |
| Never had children | Ref |  |  |
| Living with children <18 during study | -7.3 (-12.5, -2.1)** | -7.1 (-10.3, 0.1) | -5.2 (-10.5, 0.2) |
| Within Person effects |  |  |  |
| Never had children | Ref |  |  |
| Number of children remained unchanged | -5.8 (-8.1, -2.6)*** | -5.9 (-8.7, -3.2)*** | -4.5 (-7.3, -1.6)** |
| First child/ Additional children | -6.0 (-9.1, -2.9)*** | -6.1 (-9.2, -3.1)*** | -5.1 (-8,3, -1.9)** |
| Fewer children (aged < 18yrs) living at home | -4.5 (-8.8, -1.8)** | -5.3 (-8.9, -1.8)* | -4.3 (-7.9, -0.6)* |
| **Change in employment status** |  |  |  |
| Between-person effects |  |  |  |
| Not employed full-time during the study period | Ref |  |  |
| Employed full-time during study period | -6.6 (-8.2, -5.2)*** | -5.9 (-7.3, -4.4)*** | -4.1 (-6.0, -2.4)*** |
| Within Person effects |  |  |  |
| Employed full-time during the study period | Ref |  |  |
| Remain part-time/not working | 0.1 (-1.7, 1.9) | -0.2 (-4.3, 1.7) | -1.9 (-4.4, 0.6) |
| Increase their work hours | -4.2 (-6.4, -2.1)*** | -4.3 (-6.4, -2.3)*** | -5.2 (-7.9, -2.6)*** |
| Reduced their work hours | -3.1 (-5.5, -0.7)** | -3.2 (-5.6, -0.8)* | -3.6 (-6.6, -0.6)* |

Abbreviation: β= beta, CI= confidence interval, ***p-value= <0.001, **p-value= <0.01, *p-value= <0.05,

Model 1: Change in parental status and sitting; adjusted for time

Model 2: Change in parental status and sitting; adjusted for time, baseline age, education status, health status and area of residence

Model 3: Change in parental status and sitting; adjusted for time, change in employment (Between person / Within person,), and baseline age, education status, health status and area of residence

Model 1: Change in employment status and sitting; adjusted for time

Model 2: Change in employment status and sitting; adjusted for time, baseline age, education status, health status and area of residence

Model 3: Change in employment status and sitting; adjusted for time, change in number of children (Between Person/Within Person), baseline age, education status, health status and area of residence

**Supplementary Table 3. Mixed-effects negative binomial regression estimates of between-person and within-person changes in TV viewing associated with changes in parental status and employment status over five years (2007/08-2011/13)**

|  | **TV viewing(hours/week)** | | |
| --- | --- | --- | --- |
|  | **Model 1** | **Model 2** | **Model 3** |
|  | **IRR (CI)** | **IRR (CI)** | **IRR (CI)** |
| **Change in parental status** |  |  |  |
| Between-person effects |  |  |  |
| Never had children | Ref |  |  |
| Living with children <18 during study | 0.85 (0.66, 0.96)* | 0.78 (0.64, 0.90)** | 0.77 (0.64, 0.93)* |
| Within Person effects |  |  |  |
| Never had children | Ref |  |  |
| Number of children remained unchanged | 0.92 (0.83, 1.02) | 0.85 (0.77, 0.95)** | 0.81 (0.71, 0.91)** |
| First child/ Additional children | 0.87 (0.78, 1.06)* | 0.85 (0.75, 0.95)** | 0.80 (0.80, 1.01)** |
| Fewer children (aged < 18yrs) living at home | 0.95 (0.83, 1.09) | 0.87 (0.76, 1.00) | 0.83 (0.78, 1.02)* |
| **Change in employment status** |  |  |  |
| Between-person effects |  |  |  |
| Employed full-time during the study period | Ref |  |  |
| Not employed full-time during study period | 1.01 (0.94, 1.06) | 1.00 (0.97, 1.11) | 1.00 (0.92, 1.08) |
| Within Person effects |  |  |  |
| Employed full-time during the study period | Ref |  |  |
| Remain part-time/not working | 1.04 (0.97, 1.15) | 1.04 (0.96, 1.11) | 1.10 (1.01, 1.20)* |
| Increase their work hours | 0.96 (0.87, 1.03) | 0.94 (0.86, 1.02) | 1.04 (0.93, 1.15) |
| Reduced their work hours | 1.10 (1.01, 1.20)* | 1.16 (0.99, 1.18) | 1.25 (1.11, 1.40)*** |

Abbreviation: IRR= ratio of mean, CI= confidence interval, ***p-value= <0.001, **p-value= <0.01, *p-value= <0.05,

Model 1: Change in parental status and TV time; adjusted for time

Model 2: Change in parental status and TV time; adjusted for time, baseline age, BMI, education status and smoking status

Model 3: Change in parental status and TV time; adjusted for time, change in employment (Between Person/Within Person), and baseline age, BMI, education status and smoking status

Model 1: Change in employment status and TV time; adjusted for time

Model 2: Change in employment status and TV time; adjusted for time, baseline age, BMI, education status and smoking status

Model 3: Change in employment status and TV time; adjusted for time, change in parental status (Between Person/Within Person), and baseline age, BMI, education status and smoking status

**Supplementary Table 4. Mixed-effects negative binomial regression estimates of between-person and within-person changes in computer time associated with changes in parental status and employment status over five years (2007/08-2011/13)**

|  | **Computer time (hours/week)** | | |
| --- | --- | --- | --- |
|  | **Model 1** | **Model 2** | **Model 3** |
|  | **IRR (CI)** | **IRR (CI)** | **IRR (CI)** |
| **Change in parental status** |  |  |  |
| Between-Person effects |  |  |  |
| Never had children | Ref |  |  |
| Living with children <18 during study | 0.72 (0.52, 0.98)* | 0.73 (0.53, 0.99)* | 0.85 (0.61, 1.12) |
| Within-Person effects) |  |  |  |
| Remain with no children | Ref |  |  |
| Number of children remined unchanged | 0.61 (0.50, 0.73)*** | 0.67 (0.56, 0.83)*** | 0.73 (0.55, 0.84)** |
| First child/ Additional children | 0.57 (0.47, 0.69)*** | 0.54 (0.44, 0.66)*** | 0.56 (0.43, 0.66)*** |
| Fewer children (aged <18yrs) living at home | 0.53 (0.43, 0.67)*** | 0.64 (0.50, 0.82)*** | 0.66 (0.49, 0.80)*** |
| **Change in employment status** |  |  |  |
| Between-Person effects |  |  |  |
| Employed full-time during the study period | Ref |  |  |
| Not employed full-time during the study period | 0.57 (0.51, 0.63)*** | 0.61 (0.54, 0.67)*** | 0.68 (0.60, 0.78)*** |
| Within-Person effects |  |  |  |
| Remain full-time | Ref |  |  |
| Remain part-time/not working | 0.88 (0.79, 0.98)* | 0.87 (0.78, 0.97)** | 0.77 (0.66, 0.90)** |
| Increase their work hours | 0.87 (0.76, 0.99)* | 0.87 (0.77, 0.99)** | 0.81 (0.68, 0.96)* |
| Reduced their work hours | 0.70 (0.61, 0.80)*** | 0.65 (0.57, 0.75)*** | 0.70 (0.58, 9.86)** |

Abbreviation: IRR= ratio of mean, CI= confidence interval, BP= between-person, WP= within-person, ***p-value= <0.001, **p-value= <0.01, *p-value= <0.05,

Model 1: Change in parental status and computer time; adjusted for time

Model 2: Change in parental status and computer time; adjusted for time, baseline age, education, and area of residence

Model 3: Change in parental status and computer time; adjusted for time, change in employment (Between Person/Within Person) and baseline age, and education

Model 1: Change in employment status and computer time; adjusted for time

Model 2: Change in employment status and computer time; adjusted for time, baseline age, education, and area of residence

Model 3: Change in employment status and computer time; adjusted for time, number of children (Between Person/Within Person) and baseline age, and education
